# ECD-UY, detailed household electricity consumption dataset of Uruguay

**DOI:** 10.1038/s41597-022-01122-x

**Published:** 2022-01-20

**Authors:** Juan Chavat, Sergio Nesmachnow, Jorge Graneri, Gustavo Alvez

**Affiliations:** 1 Universidad de la República, Montevideo, Uruguay; 2 UTE, Montevideo, Uruguay

**Keywords:** Energy supply and demand, Energy modelling, Energy modelling

## Abstract

This article introduces a dataset containing electricity consumption records of residential households in Uruguay (mostly in Montevideo). The dataset is conceived to analyze customer behavior and detect patterns of energy consumption that can help to improve the service. The dataset is conformed by three subsets that cover total household consumption, electric water heater consumption, and by-appliance electricity consumption, with sample intervals from one to fifteen minutes. The datetime ranges of the recorded consumptions vary depending on the subset, from some weeks long to some years long. The data was collected by the Uruguayan electricity company (UTE) and studied by Universidad de la República. The presented dataset is a valuable input for researchers in the study of energy consumption patterns, energy disaggregation, the design of energy billing plans, among other relevant issues related to the intelligent utilization of energy in modern smart cities.

## Abstract (summary)

**Table:** 

Measurement(s)	Electricity
Technology Type(s)	smart meter • clamp meter • sonoff device
Factor Type(s)	temporal interval
Sample Characteristic - Location	Uruguay

Machine-accessible metadata file describing the reported data: 10.6084/m9.figshare.16965400

## Background & Summary

Worldwide, electricity consumption of residential household showed an uninterrupted growth in the last fifty years^1^. It is expected that in 2050 the demanded electricity consumption doubles the one recorded at 2010^2^. Providing the future demanded electricity supply is a challenge and many investigations are taking place in this sense^3–6^.

In Uruguay, electricity is provided by the state-owned electric company, Administración Nacional de Usinas y Trasmisiones Eléctricas (UTE). Uruguay has been recognized as one of the top countries with the most developed and used renewable energy sources. Uruguayan population is 3.4 million people, 1.3 million of them living in its capital, Montevideo. Electrification is considered universal, counting 99.8% of total areas (rural and urban)^7^. By July 2019, UTE provided electricity to 1,498,164 customers countrywide, of which 90.5% are residential^8^. The company provides a monthly average of 228 kWh per residential customer, 246 kWh in Montevideo and 216 kWh in the rest of the country^9^, for a total value of 368.5 GWh to residential customers. In Uruguay, 87.3% of residential households have electric water heaters (mainly for showers)^10^. The consumption of this appliance, which is fully manageable and has a high potential for thermal storage, represents approximately a third of the electrical consumption of all homes. In turn, the electrical matrix has diversified using renewable resources such as wind, solar, biomass energy, whose energy generation depends on weather conditions. This scenario allows implementing a proper management of the generated energy, making use of the potential of thermal storage in electric water heater of residential customers (see a description of this possible application on section ‘Applicabity’, at the end of this article).

A joint research project between UTE and the national university, Universidad de la República, was proposed to study the electricity consumption patterns of residential customers. In this context, the main motivations to create the presented dataset are related to study those patterns, detect similarities and anomalies, and be used as input of intelligent algorithms for planning, designing a recommendation system for citizens, and improve the overall quality of the electric service.

**Fig. 1:**
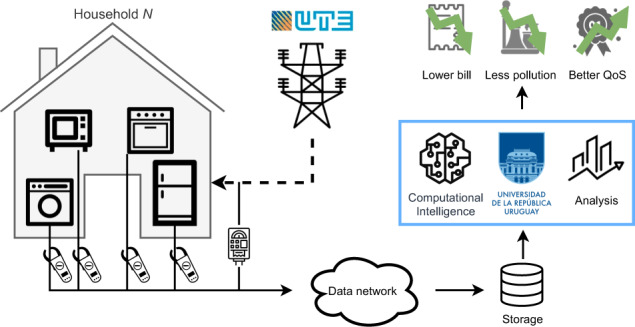
Schematic overview of the designed system for collecting and processing household electric consumption data.

The systems designed to collect the data use different devices. The total household consumption is obtained from clamp meters or directly from smart meters (if available), while the dissaggregated consumption of the appliances is obtained by clamp meters or plug-in meters. Figure 1 shows a schematic overview of the data collection systems and the main processes involved.

The presented dataset, named *ECD-UY* after *Electricity Consumption Data set of UruguaY*, is divided into three consumption subsets and a customers information set. Regarding consumption data, the first subset consists of the total household consumption obtained from smart meters of 110,953 customers countrywide. The sample interval of the records is fifteen minutes, and the monitoring date range starts on 1^*st*^ January 2019 and ends on 3^*rd*^ November 2020. The consumption data is referred as *aggregated*, since the consumption of individual appliances is not reported, but the overall household consumption instead. The second subset consists of the *electric water heater consumption* of 268 households, from different cities in Uruguay. The sample interval of the records is one minute and the date range of consumption records is from 2^*nd*^ July 2019 to 26^*th*^ October 2020. The third consumption subset consists of two relevant data: the total aggregated consumption records of nine households in Montevideo, and the disaggregate consumption of a set of appliances in each household (e.g., lamps, fridges, air conditioner, etc). The sample interval is one minute and the date range is from 27^*th*^ August 2019 to 16^*th*^ September 2019. The set of customers information includes data about customers, contracted service, and geolocalisation. Table 1 summaries the characteristics of each consumption subset in the ECD-UY dataset.

**Table 1:** Summary of the four subsets contained in ECD-UY.

subset	households	total consumption	dissagregated consumption	period	start date*	end date*
total household consumption	110,953	yes	no	15 min.	01/01/2019	03/11/2020
electric water heater	268	yes	—	1 min.	2/07/2019	26/10/2020
appliances consumption	9	yes	yes	1 min.	27/08/2019	16/09/2019
customers information	110994	—	—	—	—	—

*Periods may vary depending on the customer.

Several datasets of energy consumption have been recently made available to the research community. Some well-known energy datasets are:

- UK-DALE, including disaggregated consumption data from five UK households^11^;
- REDD, including disaggregated consumption data from six households in New Jersey, USA^12^;
- AMPds2, including electricity, water, and natural gas consumption of a single house located in Vancouver, Canada^13^;
- DEDDIAG, including electricity disaggregated consumption of appliances from 15 households in Germany^14^;
- IDEAL, including information about electricity, gas, and contextual data from 255 households in UK^15^;
- ENERTALK, including aggregated and disaggregated electricity consumption data from 22 houlseholds in Korea^16^.

ECD-UY is the only public dataset describing residential electricity consumption with low-interval records in Uruguay, also the first available in its type in Latin America.

## Methods

This section describes the methods applied for data collection, data communication, and pre-processing/cleansing. The information is reported for each collected subset.

### Data collection

Different data collection processes were applied for each subset. The main details of the collection process, devices, and methods are reported next.

#### Total household consumption

Data of total household consumption collection was collected by the telemetry system of UTE. This system consists of smart meters installed in customers scattered around different Uruguayan cities, covering 40% of the total residential customers (approximately 600,000 out of the 1,498,164 households), at the moment of writing this article (October, 2021). The goal of the company is reaching a coverage of 100% of customers within the next two years. The deployment of smart meters started in southern cities (Montevideo, Canelones, Maldonado, and Colonia), and continued to other cities along the country. As of October 2021, 600,000 smart meters have been installed. Actually, the installation of smart meters is part of the main operation of the company, within the development of a new smart grid infrastructure. Approximately 86% of the installed smart meters use the 3 G network for transmitting the measured data. In turn, 10% use optic fiber communications and 4% use Power Line Communications (PLC).

**Fig. 2:**
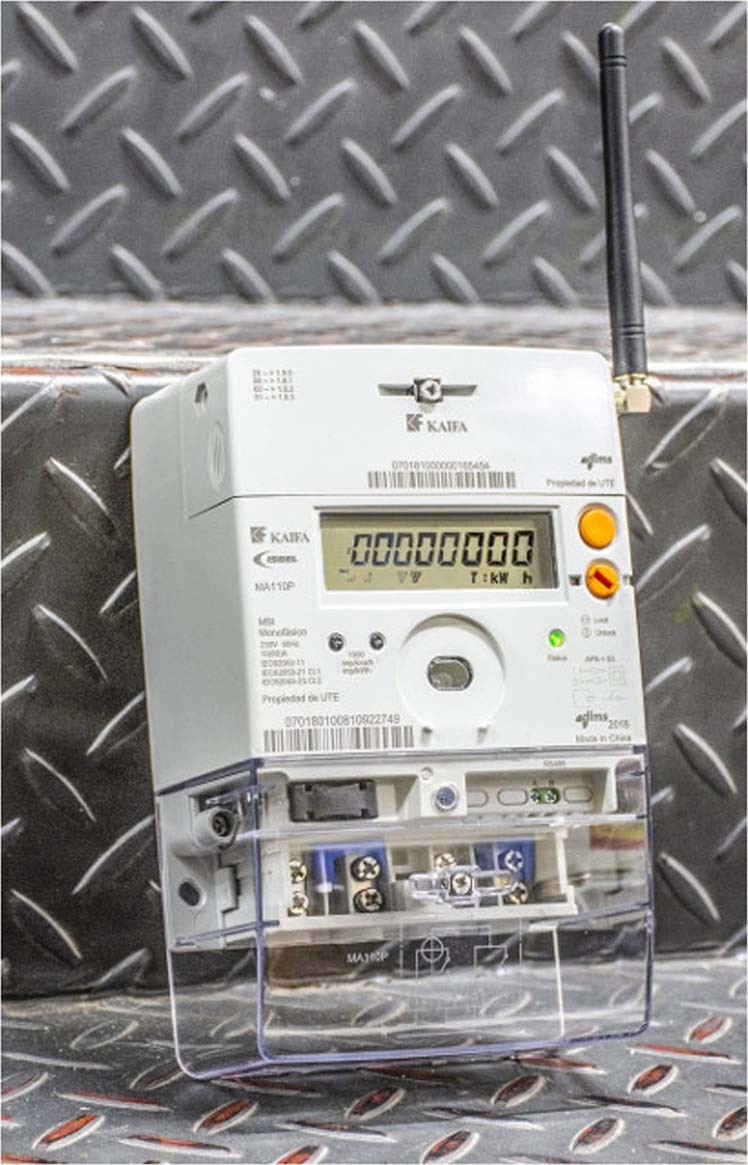
Smart meter Kaifa, model MA110P, installed by the Uruguayan electricity company, UTE (image by UTE, https://portal.ute.com.uy/medicion-inteligente).

The smart meters used in the deployment are KAIFA models MA110P (the most used, depicted in Fig. 2), MA309P, and MA309D. All of them follow standards IEC 62052–11, 62053–11/21/23, and 62056–21/46/53/61/62. The devices allow measuring active and reactive energy, voltage and current, frequency, and offers 9,600 bps modem communication, PLC/RF/GPRS/3 G, RS-485. The measurement reporting period is configurable in ranges of 5, 10, 15, 30 or 60 min (the default value is 15 min). More specifications about these devices can be found at http://kaifametering.com/.

The interval for data transmission was set to 15 min, the default value that KAIFA meters bring from origin, since this period is useful for characterization and billing purposes. This subset does not have the level of detail of the other two subsets (i.e., electric water heater consumption and disaggregated electricity consumption by appliance), which are obtained with a frequency of one minute, but it has the electricity consumption of at least ten times more dwellings. Gathering the total household consumption with smaller frequency (e.g., one minute) would imply handling with a very large volume of information. In turn, it would require a greater infrastructure of the company database. This has not been considered yet in the context of the pilot plan under development.

#### Electric water heater consumption

Electric water heaters considered in the study are all of accumulation type by electric resistance. Consumption data were collected from a set of 268 households of customers who were offered economic incentives to participate. The offered incentives were part of a commercial plan aimed at studying electricity consumption patterns. The electric water heater appliance was chosen for the study because it is one of the most energy-intensive household appliances in Uruguayan households. Customers participating in the commercial plan were properly selected to avoid bias: i) from representative substations with average historical consumption, average income and socio-economic level, and standard water heaters; and ii) from other substations to properly sample other relevant consumptions, electric water heaters, and tariff classes in the eight departments/districts considered in the study. The economic incentive offered to customers was 6 USD per month (corresponding to the price of 50 kWh charged according to the standard tariff). Users were also provided with the additional functionality of being able to remotely control their water heater, via a web/mobile application. The households of customers participating in the plan were located in different districts of eight provinces (departments) in the country (Canelones, Montevideo, Salto, Paysandú, Maldonado, Río Negro, Colonia, and San José) where the company installed a meter device and advised on the operation of it. The presented subset includes georeferenced consumption of customers located in three departments: Montevideo, Canelones and Paysandú. These locations provide a proper representative sample of residential electricity consumption in Uruguay.

**Fig. 3:**
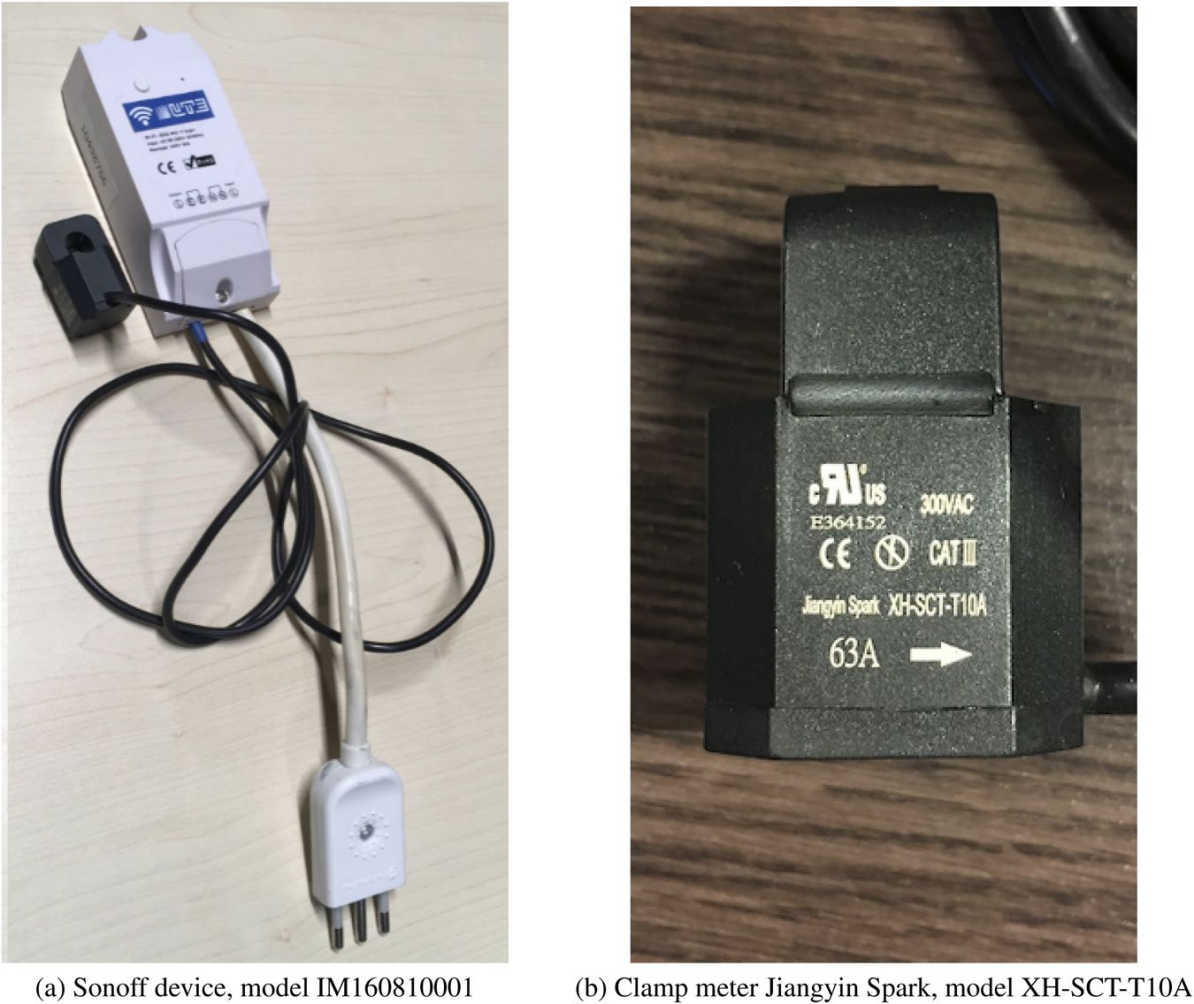
Devices installed by the Uruguayan electricity company, UTE (images by UTE).

Devices installed by UTE, used for measuring and transmitting the electricity consumption records, were smart switches (Sonoff brand, IM160810001 model) that consist of a plug-in power meter connected to the plug of the electric water heater. The used model includes a HLW8012 single phase energy monitor chip and, measuring a maximum wattage of 3,500 W, a voltage range between 90–250 V AC, a maximum current of 15 A, and its wireless frequency range is 80–160 MHz under the standard IEEE 802.11 b/g/n. The Sonoff device is presented in Fig. 3(a).

#### Disaggregated electricity consumption by appliance

The data of electricity consumption by appliance was collected in a pilot plan developed in nine households located in Montevideo and Canelones. The monitored appliances included microwave, washing machine, fridge, water heater, oven, air conditioner, dehumidifier, and tumble dryer. The selection and labelling of the appliances were carried out by the occupants themselves. Thus, appliance selection criteria were totally under the control of the customers and not of the company. Three of the dwellings were apartments while the rest were houses. In average, the number of occupants per households was 3.0, 66.6% adults and 33.4% child. Clamp meters Jiangyin Spark, model XH-SCT-T10A, were used to measure the total aggregated consumption and the consumption of each monitored appliance, with a frequency of one minute. The clamp used for measurements is presented in Fig. 3(b). Table 2 summarizes the dwelling characteristics.

**Table 2:** Dwellings characteristics of the subset of disaggregated electricity consumption by appliances.

household id	region	dwelling type	location	adults/childs
1	Montevideo	House	Urban	2/0
2	Canelones	House	Coast	2/0
3	Montevideo	Apartment	Urban	2/1
4	Montevideo	Apartment	Urban	2/1
5	Montevideo	House	Urban	2/2
6	Canelones	House	Coast	2/2
7	Montevideo	House	Urban	2/2
8	Montevideo	House	Urban	2/1
9	Montevideo	Apartment	Urban	2/0

### Communication

Collected data were transmitted to centralized data servers using different mechanisms. A description of the communication process for each subset is presented next.

#### Total household consumption

**Fig. 4:**
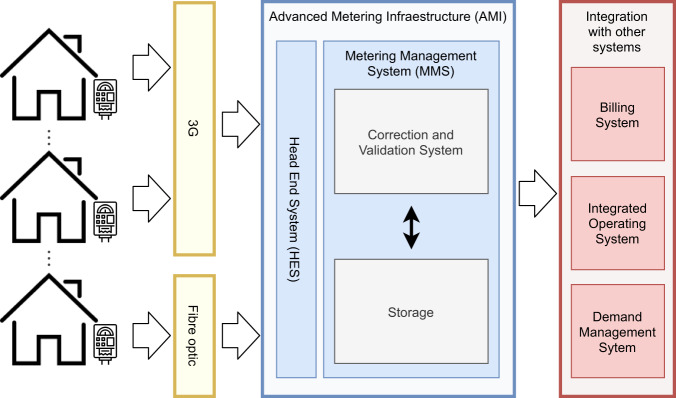
Architecture and processes of the communication system for the aggregate energy consumption dataset.

Once the aggregated consumption data is generated in each smart meter, it is transmitted to be stored in the Advanced Metering Infrastructure (AMI) of UTE. The AMI is a crucial component of modern smart grids, which is in charge of measuring the power consumption, implementing bidirectional communication between the customer and the service provider to communicate the obtained records, performing control tasks to optimize energy utilization, and implementing data management processes. The AMI is also the responsible of the communication with the smart meters and is the nexus with the billing system, the integrated operating system and the demand management system. Communication between the meters and the AMI is carried out via the 3 G communication protocol for most of the households (86%). When the household location or dwelling makes it impossible to have a 3 G connection (14%) (e.g., it is not in a coverage area), the smart meters are linked via RS-485 port to a hub connected to the fibre optic network of the National Telecommunications company (ANTEL) or PLC. The architecture and processes of the communication system for the total household consumption subset is presented in Fig. 4.

#### Electric water heater consumption

Subset records were collected using plug-in power meters and communicated by two different media: the household Internet connection or the 2 G Internet connection. Communication media are described in the following paragraphs.

In an initial deployment of the system, the plug-in power meters used the household Internet wireless connection to establish a bidirectional channel to send measurement data and receive switch-on/switch-off commands. Later on, updated models of the plug-in power meters were installed, capable of establishing a connection through a built-in 2 G modem. The update of the meters improved the robustness of the connections, thus fewer data losing during the transmission, and brought independence from the Internet connection of the customers. Both connection media secure data by establishing VPN networks. The management of the communication was carried out by a chipset integrated into the meter itself. The model of that chipset is STM32 and the software embedded in it was implemented in C language and is property of UTE.

**Fig. 5:**
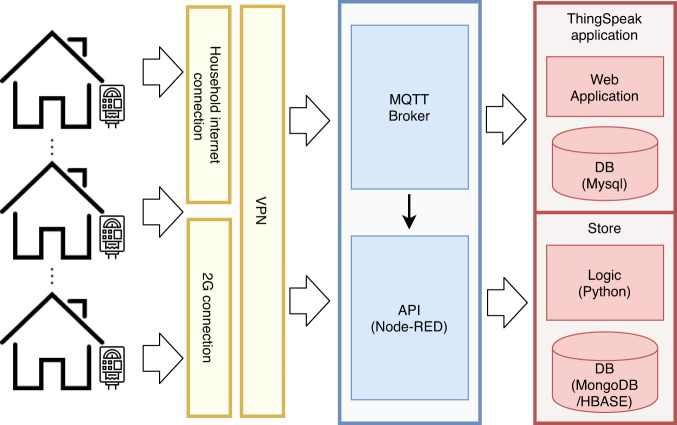
Architecture and processes of the communication system for the electric water heater consumption dataset.

Collected data was transmitted with a frequency of one minute via the lightweight MQTT network protocol. Received data was processed at the UTE infrastructure using a demand management platform implemented over the Spring Boot framework and the Java programming language. The architecture and processes of the communication system for the electric water heater consumption subset is presented in Fig. 5.

#### Disaggregated energy consumption by appliance

Clamps used for measuring electricity consumption communicate the collected recorded data to a gateway/hub inside the dwelling via the Zigbee 3.0 protocol. Once a measure is recorded, it is sent via a wireless Internet connection to a remote third-party service. The service centralizes the storage of all data received from the clamps installed in the customers and it also associate the recorded consumption with dwelling metadata. The clamps measure and send the consumption with a sample period of one minute, but in case of loss of connection, the gateway/hub counts with buffer storage able to hold up to until the connection is reestablished. Regularly, UTE downloads the updated data, as text files, from the third-party servers.

### Pre-processing and cleansing

Data were pre-processed and cleansed to be used as a common baseline for comparison of results in researches using the ECD-UY dataset. Unix scripts using different tools (e.g., awk, split, sort, uniq, etc) and three Jupyter notebooks using Python language version 3 and several utility libraries, including Pandas, Numpy, and Dask^17,18^, were implemented for the process. Data processing was performed using big data and urban informatics techniques, on the high performance computing platform of National Supercomputing Center, Uruguay (Cluster-UY)^19^.

In general, transformation methods were applied over the collected raw data in order to: i) standardize the units, date formats, column names and file names, ii) remove unnecessary columns, and iii) build unique columns from the fusion of two or more columns. The transformations applied to customers and energy consumption data are described in the following paragraphs.

#### Customers data

The data about customers, provided by UTE, consisted of three files, one for each subset. The files contained customers that did not match with electricity consumption information, i.e., there was customers information without consumption records. Useless customers information was removed from each file and then, the three files were merged and standardised into a unique file. In the case of customers from the *dissagregated energy consumption by appliance* subset, the identifiers of the customers were changed in the consumption/appliances files to avoid collisions with different customers presented in the *total household consumption* subset. Also, the leading and trailing spaces in tension and tariff columns were removed.

#### Total household consumption

At the AMI module, the data was corrected and validated and then delivered to the meter data management system. Corrections were applied in case of detecting anomalies or missing data. After being processed by the AMI, the data was available for analysis. After applying the pre-processing and cleansing process to the raw data provided by UTE, the consumption records were stored in one file per month, to get appropriate file sizes (between 780 MB and 7.1 GB). The raw data had a file to describe the meter-customer relationship. After checking that there was only one meter per customer in the consumption files, the meter identifier was replaced by its corresponding customer identifier, reducing the overall size of the subset and simplifying the file structure. Finally, records with a null value in the customer identifier were removed, and the datetime column was represented in epoch time format, which allowed achieving a significant overall size reduction (20% less size, comparing with the version of ISO-8601 datetime format).

#### Electric water heater consumption

As part of the pre-processing of the collected records, the mean power, mean voltage, and instant power of the electric water heaters were calculated using specific firmware developed (in C language) on the STM32 chips of the Sonoff meters, using the HLW8012 single phase energy monitor chip. Then, the identifier of the meter was replaced by the identifier of the corresponding customer, in those records counting with customer information. Additionally, datetime columns were formatted to epoch time format. Both changes shorten the record length, reducing the overall file sizes. Finally, consumption records that belonged to customers/meters with less than 1440 records (i.e., the number of records corresponding to one day) were removed from the subset. Pre-processing and data cleansing reduced the total file size of the subset from 6.6 GB to 2.4 GB.

#### Disaggregated energy consumption by appliance

UTE collected customer information (e.g., household census areas and department) and related it to the appliances by a meter identifier. In turn, meter identifiers are the link between appliance information and its consumption records. Several types of consumption signals (e.g., active and reactive energy, active and reactive power, etc.) were recorded for a meter at the same datetime, as different rows in the consumption collection. In order to simplify the processing of multiple rows referencing the same meter at the same time, the multiple rows were transformed into a single consumption row with multiple columns, one per type of consumption signal. For the same reason, the consumption records of the appliances and the total consumption were separated into different collections.

During the cleansing stage, appliance information and consumption records were removed due to the meter identifiers were not present in both collections (i.e., appliances information without consumption records, or consumption records without appliances information). In total, 34 appliances and 1,163,714 consumption records were removed.

## Data Records

ECD-UY is available to download from the public repository, figshare^20^. The download file contains a structure with three directories, one per subset. The directory *total-household-subset* contains all the files related to the total consumption subset, *electric-water-heater-subset* contains the files related to the electric water heater subset, and *disaggregated-by-appliance-subset* contains the files related to disaggregated energy consumption by appliance. The data files inside each directory are in the CSV common format^21^ and their columns are described in the following subsections. To reduce the amount of needed storage in the repository, large size files were compressed and presented together with reduced (and not compressed) sample of the data.

### Customers information

The information of the customers is presented in a unique file, customers.csv, for all the subsets. The information consists of an identifier, characteristics of the electricity service contracted, and geolocalisation data in four levels. The records are detailed in Table 3. Customers records are related with the rest of the files by the value of the column id

**Table 3:** Description of the records corresponding to the information of customers, present in the file customers.csv.

customers.csv		
field	*type*	*description*
id	number	Unique value to identify the household
tension	string	Voltage at which residential customers are connected to the grid (230 V or 400 V)
tariff	string	Type of contracted tariff. TCB: basic consumption; TRS: residential (simple); TRD: residential (double band); TRT: residential (triple band); TCBT: discount TCB [social assistance for low-income households]
power	number	Contracted power, in W
department	number	Department where the household is located
section	number	Censal section where the household is located
segment	number	Censal segment where the household is located
zone	number	Censal zone where the household is located

### Total household consumption

The subset of total household consumption includes files of consumption records, one per month, each one named as consumption_data_AAAAMM.csv. The text “AAAAMM” included in the filenames corresponds to the year (AAAA) and the month (MM) of the records contained in the file. Table 4 reports the details of the information provided by each record. The information of the customers and the consumption records relate to each other by the value of the id and customer_id columns, respectively.

**Table 4:** Description of records in files of the total household consumption dataset.

consumption_data_AAAAMM.csv		
*field*	*type*	*description*
datetime	string	Datetime of the record, in Epoch time format
id	number	Unique value to identify the customer
value	number	Value of active energy, in kWh

### Electric water heater consumption

The electric water heater consumption subset includes two files. On the one hand, the file consumption_data_customers.csv stores the consumption records of electric water heaters for which the customer information is available (stored in the customer information file, customers.csv). On the other hand, the file consumption_data_timers.csv stores the consumption records of electric water heaters without customer information. The conceptual separation into two files allows processing consumption data depending on the availability of information of customer, without requiring data filtering. A description of the records on each file is presented in Table 5.

**Table 5:** Description of records in files of the electric water heater dataset.

consumption_data_customers.csv		
*field*	*type*	*description*
datetime	string	Datetime of the record, in Epoch time format
customer_id	number	Unique value to identify the customer
power	number	Instant power in watts (W)
voltage	number	Instant voltage in Volts (V)
**consumption_data_timers.csv**		
datetime	string	Datetime of the record, in Epoch time format
meter_id	number	Unique value to identify the timer (meter)
power	number	Instant power in watts (W)
voltage	number	Instant voltage in Volts (V)

Only the records of the file consumption_data_customers.csv are linked with the information of customers by the value of the column customer_id in the consumption file, and the value of the column id in the customers information file.

### Disaggregated energy consumption by appliance

The disaggregated energy consumption by appliance data subset is integrated by the total aggregated consumption records plus the disaggregated consumption of different household appliances. The electricity consumption was recorded for the following appliances: air conditioner, dehumidifier, electric air heater, electric oven, electric water heatering, fridge, microwave, tumble dryer, and washing machine. It is worth clarifying that, since the customer chose the appliances to monitor, may occur that some of the appliances were present in the household but were not monitored. Three files are included in the subset: appliances.csv, appliance_consumption_data.csv, and total_consumption_data.csv. A description of the records in each file is presented in Table 6. The relationship between records of different files is given by the columns customer_id, appl_meter_id and meter_id, when applicable.

**Table 6:** Description of records in the disaggregated energy consumption by appliance dataset.

appliances.csv		
*field*	*type*	*description*
id	number	Unique value to identify the customer
appl_meter_id	number	Unique value to identify the appliance meter
appl_desc	string	Appliance description (in Spanish)
appl_type	string	Appliance type (based on the nilmtk categories^29^)
**total_consumption_data.csv**		
datetime	string	Datetime of the record, in epoc time format
meter_id	number	Unique value to identify the appliance meter
aenergy	number	Accumulated active energy in the last minute, in Wh
aenergy_ph{1,2,3}	number	Accumulated active energy in the last minute in phase 1, 2 and 3, in Wh
renergy	number	Reactive energy, in VArh
renergy_ph{1,2,3}	number	Reactive energy in phase 1, 2 and 3, in VArh
apower	number	Aactive power in W
apower_ph{1,2,3}	number	Active power in phases 1, 2 and 3, in W
rpower_ph{1,2,3}	number	Reactive power in phase 1, 2 and 3, in VArh
current_ph{1,2,3}	number	Value of the current in phase 1, 2 and 3, in A
pfactor	number	Power factor (energy efficiency)
pfactor_ph{1,2,3}	number	Value of the power factor in phase 1, 2 and 3
voltage_ph{1,2,3}	number	Value of the voltage in phase 1, 2 and 3, in V
**appliance_consumption_data.csv**		
datetime	string	Datetime of the record, in Epoch time format
meter_id	number	Unique value to identify the appliance meter
aenergy	number	Active energy, in Wh
apower	number	Active power, in W
apower_ph{1,2,3}	number	Active power in phases 1, 2 and 3, in W

## Technical Validation

This section describes sample experiments performed to support the technical quality of the ECD-UY dataset.

### Total household consumption

The total household consumption subset includes the total aggregated consumption of 110,953 households distributed in the 19 departments of Uruguay. On average, each household was monitored for 539.2 days and each day counts with 95.2 records. The validation confirmed that all households (100%) in the total household consumption dataset have the corresponding details in the customers dataset.

Regarding the number of customers, the period of days monitored, and the number of records per day, two experiments were performed. The days considered for the experiments were classified into two groups according to the following completeness criterion. The expected number of records per day is 96 (i.e., one record every 15 min). The completeness criterion states that a *complete day* has at least 95% of the expected number of records. Results indicate that more than 97% of the days have between 91 and 96 records, i.e. the vast majority of days meet the completeness criterion. Table 7 summarize the obtained results, disaggregated by the defined intervals on the number of records.

**Table 7:** Number of days per interval of number of records.

interval of records	number of days	share
(91, 96]	58,122,666	97.16%
(86, 91]	355,269	0.59%
(81, 86]	92,690	0.15%
(76, 81]	289,154	0.48%
(72, 76]	76,332	0.13%
(0, 72]	887,232	1.48%
total	59,823,343	100%

Regarding the number of customers and the number of days monitored, validation experiments were performed considering intervals of 60 days and 1 year. Using all the available days (not filtered by the completeness criterion), results showed that the 60-days interval with more customers ranges from 480 to 540 days and that 98.8% of the customers count with several monitored days in the yearly interval from 365 to 690 days. When considering only those days that meet the completeness criterion, the 60-days interval with more customers remains the same, as well as the yearly interval but with a share of 97.5% of customers. On average, the number of days per customer drops from 539.2 to 525.2 when filtering by the criterion.

**Fig. 6:**
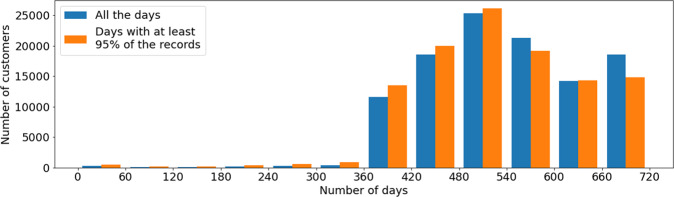
Histogram of customers with recorded consumption (all days and complete days, i.e., at least 95% of records).

In experiments using days that meet the completeness criterion, the total number of customers decreased from 110,953 to 96,565, mainly explained by a group of customers without even a day that meets the criterion. Detailed results on the experiments using 60-days intervals are reported in Table 8, and Fig. 6 shows a histogram that relates the number of days and the number of customers. The table and the histogram shows, side by side, the results of the experiments when using all days and only those days that meet the completeness criterion.

**Table 8:** Number of days by customer, for all days and complete days (at least 95% of energy consumption records).

interval of days	all days		complete days	
	customers	share	customers	share
(660, 690]	18,400	16.58%	0	0.00%
(600, 660]	14,105	12.71%	14,820	15.35%
(540, 600]	21,010	18.94%	19,032	19.71%
(480, 540]	25,643	23.11%	26,148	27.08%
(420, 480]	18,630	16.79%	20,069	20.78%
(360, 420]	11,870	10.70%	13,768	14.26%
(0, 360]	1,294	1.17%	2,728	2.83%
*total*	11,0952	100%	96,565	100%

For further validation, the annual average residential electricity consumption according to the dataset was compared with the value reported by the electric company^9^. Very close average values were obtained: 3,132 kWh according to the dataset and 3,012 kWh according to the technical report by the electric company. The small difference (i.e., just 3.8%) validates the quality of the provided data and also demonstrates that the considered households are representative of the typical electricity consumption for an Uruguayan household. Furthermore, we also crossed the information from the total household consumption subset and the customers subset and validated that just a very low percentage (0.20%) of total consumption records exceed the contracted power by the customer.

**Fig. 7:**
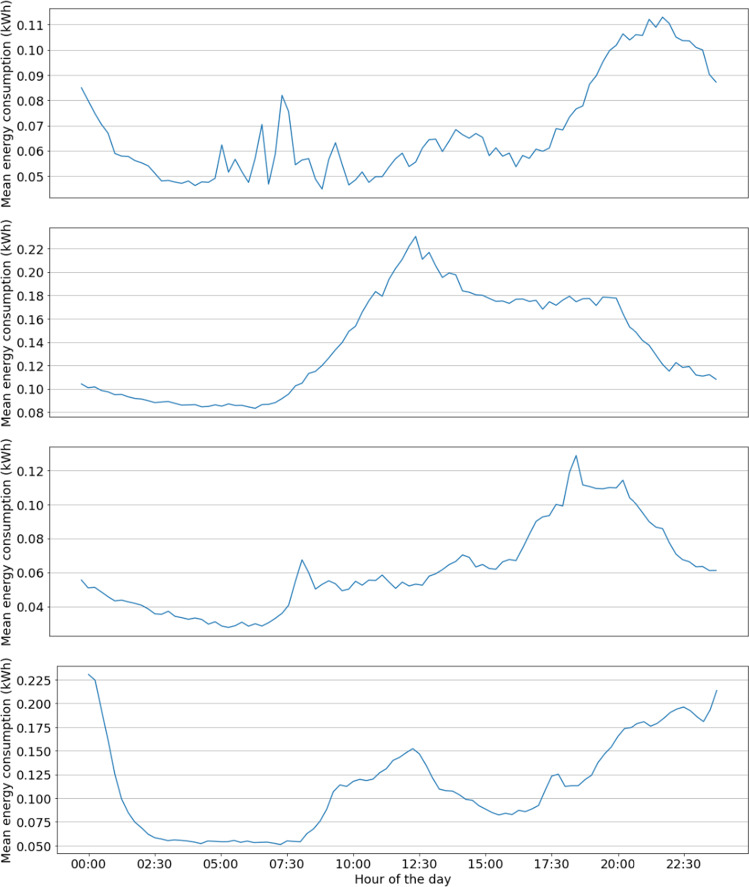
Mean energy consumption of one day for four customers (customer #8037 from 01/01/2019 to 03/11/2020, customer #97875 from 21/03/2019 to 07/11/2020, customer #109846 from 01/01/2019 to 03/11/2020, and customer #110088 from 01/01/2019 to 02/11/2020).

**Fig. 8:**
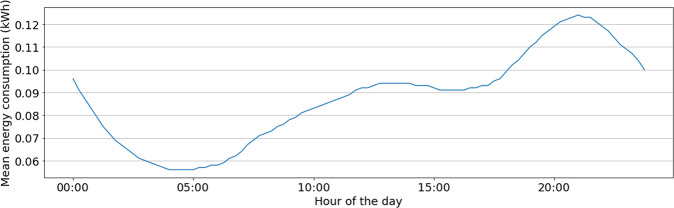
Mean energy consumption for a day.

To properly illustrate relevant examples about the quality of records and their usefulness for the analysis, Fig. 7 presents the mean energy consumption discretized in 15-minutes intervals, for four representative customers. Graphs show that the minimum consumption is during the night and some peaks are experienced mainly around the midday and at the end of the day. Different consumption profiles are detected for each customer, but when considering all customers in the subset as a whole, data in Fig. 8 allows concluding that they follow a global consumption pattern: a valley exists during late-night hours, with a minimum consumption value around 4:00 AM), and two energy consumption peaks are recognised during the day, the lowest at midday, and the highest at around 9:00 PM. These results validate the consistency of records in the total consumption dataset with standard national load profiles reported by the Electric Company^9^.

### Electric water heater consumption

For the subset of electric water heaters consumption, the validation confirmed that 166 households (62%) have the corresponding details in the set of customers. The technical validation evaluates the subset together with the corresponding total consumption, filtered by date range first and by the customer identifier then, resulting in the consumption of 135 customers.

For the technical validation, records were filtered by percentile criteria trying to avoid data anomalies (e.g., exceptionally high consumption values). First, statistics and percentiles were calculated and studied to detect the outlier values, and then the consumption values detected as outliers were removed. Two different criteria were applied to remove anomalous consumption records, depending on the dataset. For the total aggregated consumption, the applied criterion was transversal to all households: the same consumption limit value (2.186 kWh, corresponding to the 99^*th*^ percentile of the consumption values) was used to filter every record. On the other hand, for the electric water heater consumption, the threshold value applied to filter consumption records was calculated as the 97^*th*^ percentile of the consumption values per household, allowing to preserve the characteristics of each of each case (e.g., its climate context, the water heater model, etc.).

**Fig. 9:**
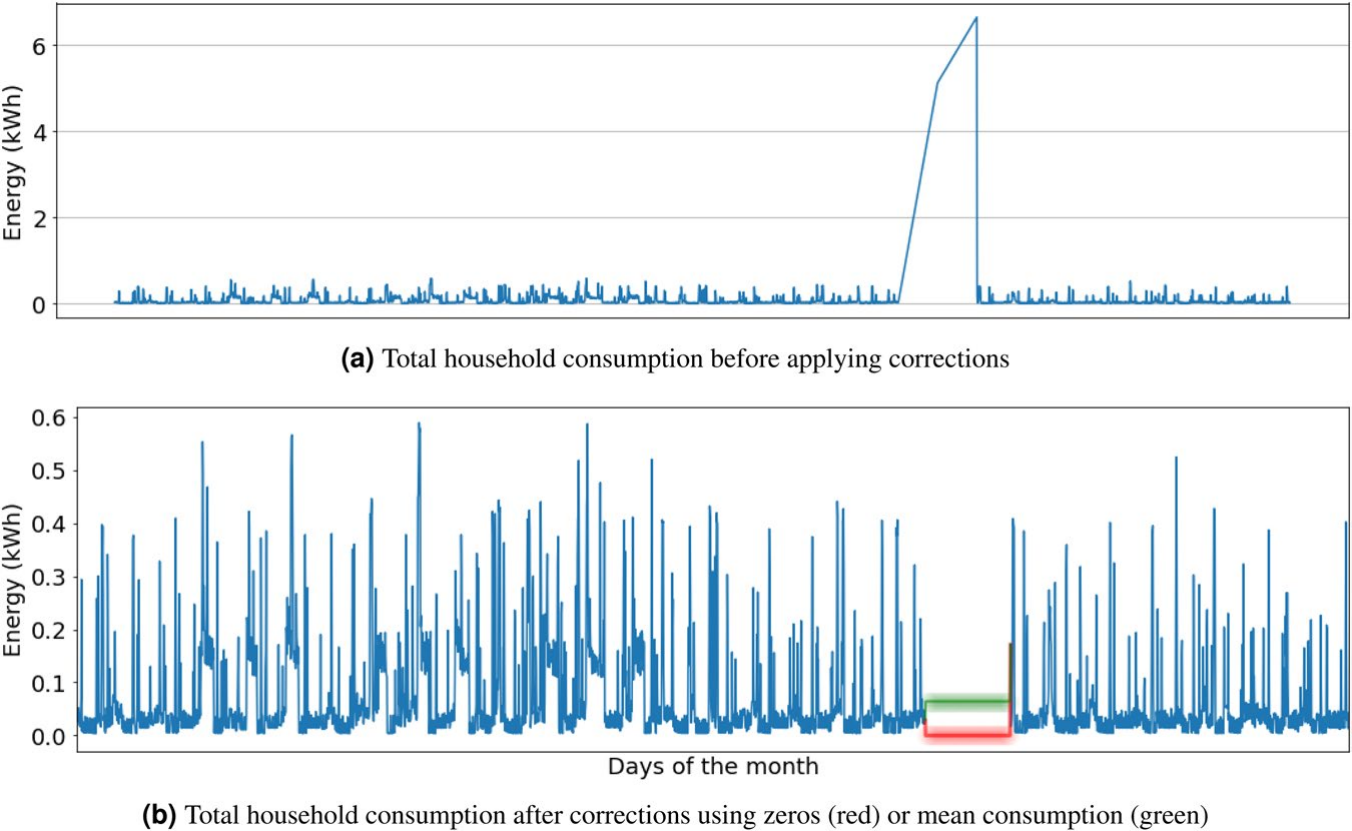
Example of one month of total household consumption with outliers and missing values, and its subsequent correction, for customer #69806.

As part of the validation, Fig. 9 shows the total consumption of one month (September 2019), for one customer (#69806), before and after refilling the detected gaps and outlier records. That is a case where data was missed for a long period. Instead, only two exceptionally high values were recorded. The missing period and the between values were filled/refilled with zeros (on red color) and with the mean energy consumption value (green). The mean length of the detected gaps for the electric water heater is 4.3 records, corresponding to 4 minutes and 19 seconds.

**Fig. 10:**
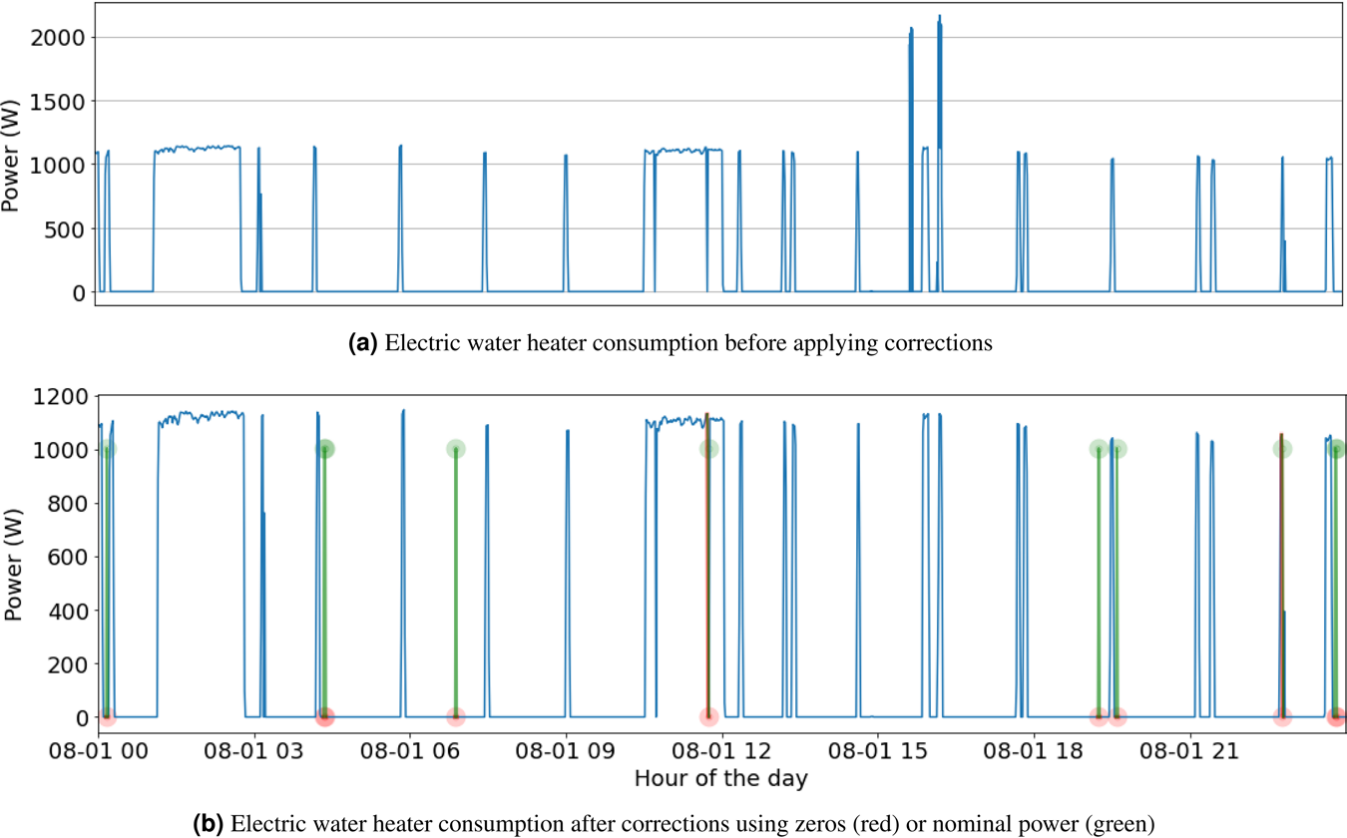
24-hours sample of electric water heater consumption for customer #115609 and two possible corrections for outlier values: set outliers to zero (red circles), and set outliers to the nominal power value of the water heater (green circles).

Likewise, Fig. 10 shows the electric water heater consumption of one representative day (August 1st, 2019), for one customer (#115609), before and after refilling the detected outlier records. In this case, no data was missing, but exceptionally high values were recorded. Since only 0.07% of the total records are detected as high values (i.e., over 2000W as reported in Fig. 10), we conjecture that those high values correspond to possibly misrecorded or wrongly transmitted records. Two possible criteria are applied for correcting high consumption values in Fig. 10: set them to zero (red circles), and set them to the nominal power value of the water heater (green circles).

Depending on the data purpose of use, anomalies can be ignored or treated in different ways. The decision on how to treat the data was left to the final user, so the result of the data cleaning described for this technical validation was not included as part of the presented dataset.

**Fig. 11:**
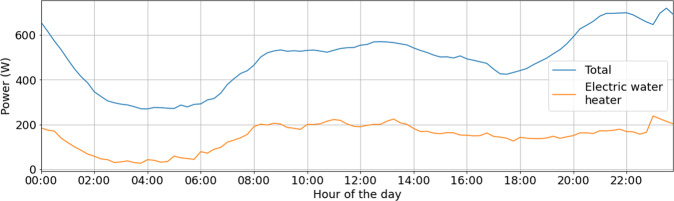
Mean total and electric water heater power consumption for a day, using the 135 households that counts with both kinds of consumption. Used period: from 15/07/2019 to 30/11/2019.

After data cleansing, the mean power consumption was calculated for periods of 15-minutes in a day. Results showed that the electric water heater has the most relevant share of the total household consumption. On average, it represents 27% of total consumption, reaching 35% during peak hours. Figure 11 shows the mean total and electric water heater power consumption for a day, highlighting the important contribution of electric water heater consumption to the total consumption. The figure was processed using the records of the 135 houses that counts with both kinds of consumption, in a period from July 7th of 2019 to November 11^st^ of 2019.

**Fig. 12:**
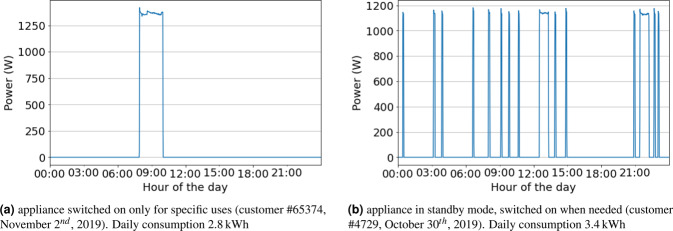
Example of electric water heater consumption patterns.

Regarding the consumption of the electric water heater, two basic patterns were identified. The first pattern shows appliances on only for those moments when hot water is needed (e.g., shower). This case is observed in the sample graphic in Fig. 12a. This pattern is also related to households with highly efficient electric water heaters appliances, which avoid standby losses. The second pattern is related to electric water heaters in standby mode during all day, with periodic consumption peaks. The water heater is automatically switched on several times a day (for short periods of a few minutes) to preserve water temperature. Figure 12b presents a sample consumption graphic for a water heater that meets this consumption pattern.

### Disaggregated energy consumption by appliance

The validation confirmed that all households (100%) in the disaggregated consumption by appliance dataset have the corresponding details in the customers dataset.

Recording periods of household appliances consumption lasted on average 19 days. During that time, data gaps (consecutive missing records) and outlier values were recorded, mainly due to meter failures and connection issues. Gaps duration depended on the appliance, ranging from one to almost seven hours. Table 9 presents detailed information about the recording and gaps duration, disaggregated by appliance.

**Table 9:** Detailed information about the recording duration and data gaps.

appliance	# appliances	mean # gaps	mean gap duration	mean recorded duration
electric air heater	2	177.0	01:50:59	19 days 19:05:00
electric oven	1	120.0	01:07:33	19 days 19:05:00
tumble dryer	1	116.0	01:40:46	19 days 19:05:02
washing machine	5	114.0	02:17:09	19 days 16:07:59
electric water heater	5	107.6	01:30:54	19 days 19:04:59
microwave	4	104.5	01:37:33	19 days 19:04:59
fridge	5	81.2	06:38:48	19 days 13:05:59
dehumidifier	1	74.0	03:25:36	18 days 04:41:58
air conditioner	3	73.3	01:50:51	19 days 19:04:59

To avoid including gaps and outliers during the validation, data were filtered and refilled. First, all consumption values lower than zero or greater than the 99^*th*^ percentile were treated as outliers and set to zero (one possible refilling criteria). Percentiles were calculated for each appliance to preserve the household context and the characteristics of each appliance. Finally, consumption gaps were detected and the missing values were set to zero.

**Fig. 13:**
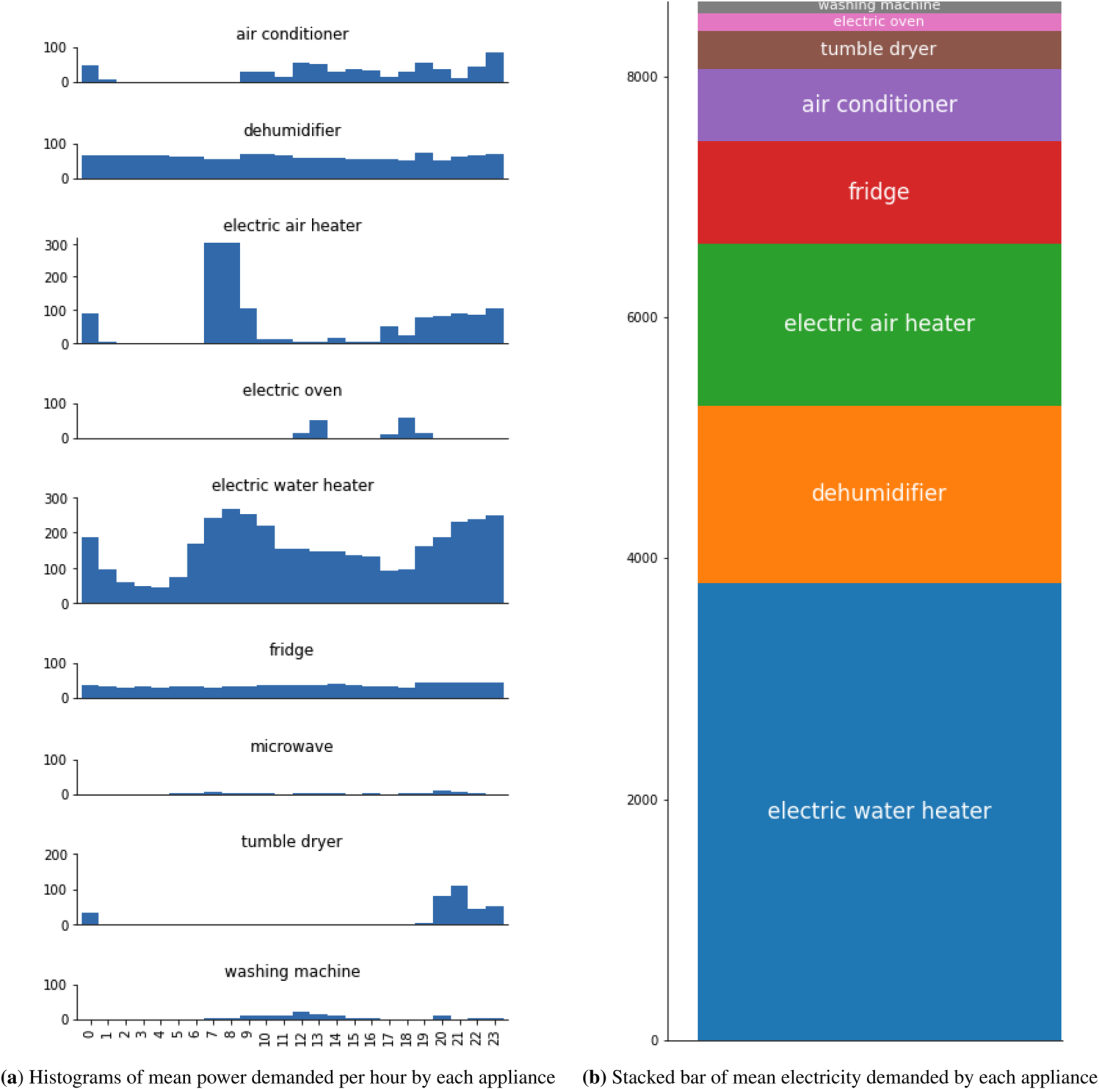
Mean power demanded by hour (histograms) and in a day (stack bar), for each appliance present in the subset.

Figure 13 presents relevant information about the power consumption of appliances in ECD-UY. Figure 13(a) shows one-hour histograms of the mean power demanded (W) by each appliance. The x-axis at the bottom of the washing machine sub-graph is also valid for the other sub-graphs. For some appliances, specific operating times are identified, e.g., for the electric oven and the washing machine, whereas for other appliances, the demand is almost constant during the day, e.g., for the fridge and the dehumidifier. Figure 13(b) shows a stack bar that accumulates the energy (Wh) demanded by each appliance in a day, reporting the average contribution of each appliance to the total household consumption. The appliances with the greatest impact on the total electricity consumption are the electric water heater, the dehumidifier, and the electric air heater.

## Usage Notes

Any software that handles CSV files can load the ECD-UY dataset. In the presented article, for processing the dataset the software used was Python version 3 and the libraries Pandas and Numpy. Loading big size files entirely in RAM memory may cause several problems that can be avoided by using the library Dask^18^. Dask can execute operations in parallel and load just the necessary data in memory. Depending on the type of processing, it may be useful to transform the dataset from CSV to Apache Parquet format^22^, which is a structured, column-oriented, compressed and binary file format that can be used for efficient processing data in Apache Hadoop and similar frameworks.

For the previously described data gaps, a rule of thumb is suggested to classify between short and long ones. If the gap duration is lower than 12 min, it may be considered short, elsewhere, it may be considered long. Short gaps are likely to be refilled by a method, e.g., interpolating or averaging the previous and forward value records. Long gaps can be considered as long periods of the appliance switched off, and therefore it would be correct to assign zero value to its consumption.

For analyzing irregular recording periods, a resample process may be applied together with value refilling criteria. A suggested resample/refilling criterion consists of creating records with regular periods and refilling with the maximum or average value (values) present in each regularized interval. An example of a resample/refilling process is implemented in the Jupyter Notebook corresponding to the technical validation of the Electric water heater subset, available to download at https://github.com/jpchavat/ecd-uy/blob/master/EWH-subset-Technical-validation.ipynb.

### Applicability of the datasets

The ECD-UY dataset is a valuable input for researchers and practitioners, and also for the electricity company, since it provides an accurate description of typical electricity consumption in Uruguay. Regarding scientific research, data from ECD-UY have been used in several projects and initiatives:

- *Design and analysis of methods for residential electricity consumption disaggregation*, with the main goal of automatically determining the appliances switched on in a household, using as input the total electricity consumption reported by a smart meter and other relevant features, by applying computational intelligence^6,23^. These techniques allow overcoming the difficulties and costs of implementing intrusive measurements, which usually are only performed in a small number of households and used as input for computational intelligence and machine learning methods.
- *Design and implementation of direct demand management strategies*, for a proper administration of electricity utilization of end consumers in a smart grid. Management is performed via modifying, reducing, or shifting the demand, to promote a better resource utilization and contributing to alleviate peak periods by shifting electricity utilization to valley (off-peak) periods, helping the grid to mitigate overloads in the electrical system. A simple and effective method is allowing the electricity company to remotely control user appliances, which is especially useful when applied to devices with thermostat that have thermal inertia, allowing a proper planning. For an effective planning by the electricity company, an accurate characterization of electricity consumption of users is needed.
- *Evaluation of demand response techniques under the smart grid paradigm*, by defining proper indexes to estimate the discomfort of users when applying an active demand management consisting of scheduled interruptions of domestic appliances (e.g., electric water heaters) when needed, to improve the overall quality of service of the electric grid^24^.
- *Designing smart recommendation systems to help users to properly plan electricity consumption to improve the cost of the bill without downgrading the quality of service*, by using real input from users, and a stochastic optimization approach to plan the utilization of domestic appliances considering (stochastic) user preferences^25,26^.

In turn, data gathered in ECD-UY is also very valuable for the electricity company, in order to study and analyze electricity consumption patterns of citizens, relating the consumption with relevant socio-demographic data and indicators^27,28^, the design of personalized electricity billing plans for different segments of the population, and the study of specific interventions to influence on the users’ behavior to achieve a rational utilization of the electric resources, among others relevant issues related to the intelligent utilization of electricity in modern smart cities.

The main lines for future work include preforming further validation experiments and conceiving additional usage applications for the reported datasets.

## Data Availability

Three Jupyter notebooks were implemented to facilitate the handling of the dataset (one notebook for each subset). The notebooks are publicly available to download from https://github.com/jpchavat/ecd-uy. For a correct execution of the notebooks, Python version 3 and the Pandas and Numpy libraries are required.
